# Transcriptome analysis of the growth-promoting effect of volatile organic compounds produced by *Microbacterium aurantiacum* GX14001 on tobacco (*Nicotiana benthamiana*)

**DOI:** 10.1186/s12870-022-03591-z

**Published:** 2022-04-22

**Authors:** Yahui Gao, Jing Feng, Jiafa Wu, Kun Wang, Shuang Wu, Hongcun Liu, Mingguo Jiang

**Affiliations:** Guangxi Key Laboratory for Polysaccharide Materials and Modifications, School of Marine Sciences and Biotechnology, Guangxi Minzu University, Nanning, 530008 China

**Keywords:** Plant growth promoting rhizobacteria (PGPR), Volatile organic compounds (VOCs), Plant growth promotion, Promotional mechanisms, Transcriptome analysis

## Abstract

**Background:**

Plant growth-promoting rhizobacteria (PGPR) release volatile organic compounds (VOCs), which promote plant growth.

**Results:**

A potential PGPR strain GX14001 was isolated from marine samples, and the VOCs produced by GX14001 significantly promoted tobacco (*Nicotiana benthamiana*) growth in a plate experiment. Based on 16S rRNA sequence alignment and physiological and biochemical characterization, GX14001 was identified as *Microbacterium aurantiacum*. Comparative transcriptome analysis was conducted between GX14001 VOCs-treated tobacco and the control; it was found that 1286 genes were upregulated and 1088 genes were downregulated. Gene ontology (GO) analysis showed that upregulated genes were involved in three biological processes: polysaccharide metabolic, polysaccharide catabolic and carbohydrate metabolic. The downregulated genes were involved in six biological processes, namely cell redox homeostasis, cellular homeostasis, carbohydrate metabolic process, homeostatic process, obsolete electron transport, and regulation of biological quality. Kyoto encyclopedia of genes and genomes (KEGG) pathway analysis showed that 190 upregulated differentially expressed genes were mainly involved in plant hormone signal transduction, phenylpropyl biosynthesis, plant–pathogen interaction, and flavonoid biosynthesis. The 148 downregulated differentially expressed genes were mainly involved in plant hormone signal transduction and the metabolism of ascorbic, aldehyde, and pyruvate acids. Further analysis revealed that many genes were differentially expressed in the metabolic pathways of plant hormone signals, which were speculated to be the main reason why GX14001 VOCs promoted tobacco growth. To further study its regulatory mechanism, we found that GX14001 promoted plant growth through auxin, salicylic acid, and gibberellin in *Arabidopsis* mutant experiments.

**Conclusion:**

The VOCs produced by *Microbacterium aurantiacum* GX14001 may promote the growth of tobacco through the auxin, salicylic acid and gibberellin pathways.

**Supplementary Information:**

The online version contains supplementary material available at 10.1186/s12870-022-03591-z.

## Background

In recent years, due to the excessive use of chemical fertilizers and pesticides, the earth’s ecological environment and agricultural soil have undergone severe changes. Therefore, reducing the application of chemical fertilizers and pesticides, and using biological fertilizers and biological control to improve production efficiency and environmental safety are required for sustainable agriculture and development [1]. The concept of plant growth-promoting rhizobacteria (PGPR) was first proposed by the American scientist J.W. Kloepper; initially, it only referred to plant growth-promoting bacteria, but in the course of research and development, the notion of PGPR has been enriched and expanded. As such, now PGPR is a group of beneficial microorganisms that can promote plant growth through various mechanisms, such as nitrogen fixation, phosphorus solubilization, disease resistance, increasing plant uptake of nutrients, adjusting phytohormone concentrations, and producing volatile organic compounds (VOCs). The microbial production of VOCs is an important growth-promoting mechanism for plants. Microorganisms, unlike promoters of plant growth, can play a role in plant growth without having direct contact with plants by producing VOCs. Microbial VOCs can promote plant growth by regulating plant hormones synthesis/transport, and inducing systemic resistance and tolerance.

### VOCs produced by PGPR regulate the synthesis/transport of plant hormones

As early as 2003, Ryu et al. found that VOCs produced by *Bacillus subtilis* GB03 could significantly promote plant growth through the cytokinin pathway [2]. Zhang et al. (2007) used “oligonucleotide microarrays” to detect the RNA transcription level of *Arabidopsis thaliana* seedlings exposed to *Bacillus subtilis* and found that the volatiles of rhizosphere bacteria GB03 could promote the growth of *A*. *thaliana* by regulating the homeostasis of auxin [3]. Huang et al. (2015) screened PGPR from the soil of a tropical rain forest and identified it as *Bacillus subtilis*. Studies have found that volatile substances of these strains can regulate the expression of 3-Indoleacetic acid (IAA)-related genes to promote the growth of *A*. *thaliana* [4]. Zhang et al. (2008) found that soil bacteria could enhance photosynthesis in *A*. *thaliana* by reducing glucose induction and abscisic acid (ABA) levels in plants. The levels of ABA biosynthetic transcripts and downstream metabolites were reduced in *A*. *thaliana* by GB03 (*Bacillus subtilis*) treatment [5]. Xie et al. (2016) found that VOCs produced by *Bacillus subtilis* JC01 and JC03 had obvious promoting effects on plants. Subsequent studies found that the VOCs produced by them have a significant upregulation effect on the genes related to the IAA signaling pathway, and the VOCs produced by JC01 have a downregulation effect on the genes related to the ABA signaling pathway, indicating that JC01 and JC03 promote plant growth mainly by regulating IAA and ABA signaling pathways [6]. Tahir et al. (2017) investigated the effects of VOCs produced by the plant growth-promoting rhizobacterium *Bacillus subtilis* SYST2 on hormone regulation and growth promotion in tomato (*Lycopersicum esculentum*) plants. Volatiles from SYST2 caused differential expression of genes related to growth and increased the auxin, gibberellin, and cytokinin content, thus promoting tomato (*Lycopersicum esculentum*) growth [7].

### Systemic resistance induced by VOCs produced by PGPR

VOCs produced by microorganisms can promote plant growth through induced systemic resistance (ISR) [8]. Ryu et al. (2004) found that *Bacillus subtilis* strain GB03 and *Bacillus amyloquefaciens* strain IN937a produced volatile compounds capable of stimulating the production of ISR in *A*. *thaliana* seedlings against *Ervinia carotovora* subsp. carotovora infection [9]. Ping et al. (2004) found that VOCs, such as butanediol and acetone, produced by certain microorganisms can induce ISR triggering in plants, and further studies on *Arabidopsis* mutants found that this growth-promoting response was related to the production of cytokinin and ethylene [10]. Rudrappa et al. (2010) showed through analysis of *Bacillus subtilis* FB17 and *A*. *thaliana* defense-impaired mutants that exogenous application of *Bacillus subtilis* to induce ethylene coupling triggers ISR and protects plants against *Pseudomonas syringae* pv. *tomato* DC3000, a process that requires the involvement of salicylic acid (SA)/ethylene (ET) and is not dependent on the jasmonic acid (JA) pathway [11]. Lee et al. (2012) found that *Bacillus polymyxa* E681 produced tridecane, which significantly induced systemic resistance (ISR) in plants [12]. Wonglom et al. (2020) found that the volatile organic compounds produced by *Trichoderma asperellum* showed antifungal activity and increased the activity of cell wall degrading enzymes, thereby promoting lettuce (*Lactuca sativa*) growth [13]. Choudoir et al. (2019) reported that VOCs produced by *Actinomycetes* were able to stimulate the growth of beneficial symbionts representing plants, and inhibiting plant pathogenic bacteria at the same time [14]. Lee et al. (2019) found that several stress-related genes were downregulated after RNA sequencing analysis of plants treated with the terpene 1-decene [15].

### System tolerance induced by VOCs produced by PGPR

Unlike ISR, which means that plants can develop systemic resistance to pathogenic microorganisms, IST refers to the ability of the strain to help plants tolerate abiotic stresses [16]. Recently, a greater number of studies have shown that PGPR and its VOCs can induce plant tolerance to abiotic stresses, including drought stress, salt stress, and nutrient deficiency, by a mechanism known as induced systemic tolerance (IST) [17]. Cho et al. (2008) found that 2R,3R-butanediol produced by *Pseudominas chlororaphis* O6 was able to induce drought tolerance in *A*. *thaliana* through a salicylic acid (SA) signaling pathway-dependent mechanism by regulating stomatal opening and closing in plants [18]. Vaishnav et al. (2015) reported that a PGPR strain *Pseudomonas simiae* AU produced VOCs that triggered tolerance to salt stress in soybean (*Glycine max*) [19]. The role of bacterial VOCs in drought tolerance in maize (*Zea mays*) was first reported by Yasmin et al. (2021), who found that VOCs produced by *Pseudomonas pseudoalcaligenes* induced tolerance in maize plants under 7-day drought stress [20]. Cappellari and Banchio (2019) found that VOCs produced by *Bacillus amyloliquefaciens* GB03 were able to promote the growth of *Mentha piperita* under salt stress. *Mentha piperita* grown in a salt-containing culture medium exposed to VOCs had better morphological characteristics and higher chlorophyll content [21]. VOCs produced by GB03 (*Bacillus subtilis*) can regulate the sodium transporter AtHKT1, so that the sodium ion level is upregulated in plant stems and downregulated in roots, resulting in low accumulation in plants, thus enabling them to survive and thrive under salt stress [5]. VOCs from *Bacillus subtilis* GB03 can increase the mRNA levels of iron absorption genes in plants and cause acidification of the plant rhizosphere, increasing the mobility of iron in plants and helping them to increase iron absorption [22].

Over the years, the studies on plant growth promotion by VOCs produced by PGPR mainly focus on *Bacillus*, *Pseudomonas*, *Actinomyces* and *Trichoderma*, but the studies on *Microbacterium aurantiacum* are few. In this study, we screened a strain of *Microbacterium aurantiacum*, named GX14001, which could significantly promote plant growth by VOCs. We performed transcriptome analysis of tobacco treated with GX14001 to analyze the growth-promoting pathway. In addition, combined with *Arabidopsis* mutants, the signal pathway and mechanism of GX14001 VOCs promoting tobacco growth were discussed.

## Results

### VOCs from GX14001 promote tobacco growth

**Fig. 1:**
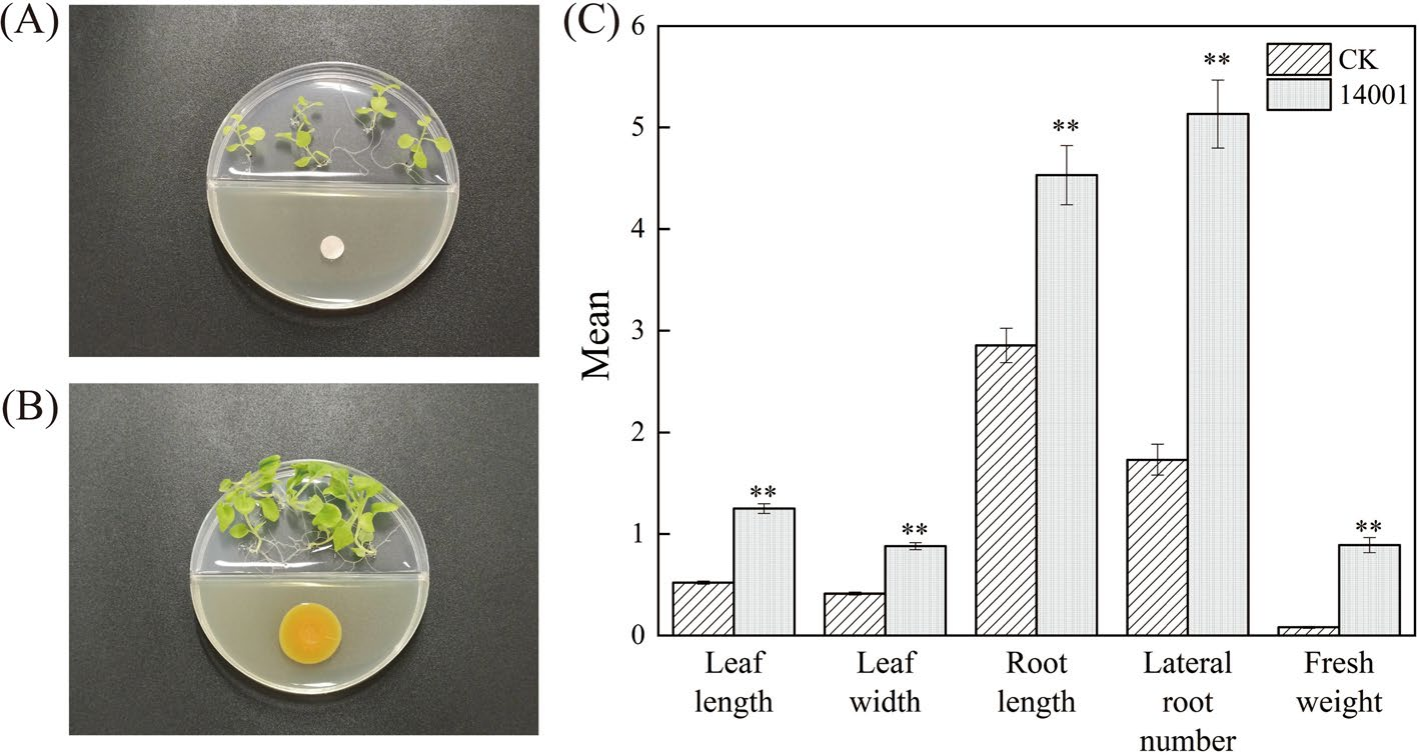
**A** The I-plate was used to co-culture tobacco seedlings and sterile water culture without physical contact; **B** The I-plate was used to co-culture tobacco seedlings and *Microbacterium aurantiacum* culture without physical contact; **C** Growth promoting effect of GX14001-VOCs on tobacco. CK was non-treated with bacteria and GX14001 was bacterial-treated with GX14001-VOCs. "*" refers to significant difference between treatments, *P* < 0.05, "**" refers to *P* < 0.01. The unit of leaf length and width and root length is cm; the unit of fresh weight is g; the unit of lateral root number is number

To detect whether GX14001 VOCs affect plant growth, tobacco seedlings were co-cultured with GX14001 on a plate divided into two sections (I-plate) at 25 °C for two weeks. Compared with the control, the fresh weight, root length, lateral root number, leaf length and leaf width of tobacco plants treated with GX14001 VOCs showed significant growth-promoter effects. As shown in Fig. 1, the experimental results showed that the VOCs produced by GX14001 significantly promoted the growth of tobacco (*P* < 0.01).

### Physiological–biochemical identification of *Microbacterium aurantiacum*

This strain was Gram stain positive, oxidase and peroxidase positive, aerobic, non-motile, and non-spore-forming. After 2 d of growth at 30 °C, the colonies on the plate were round, convex, and orange. It can be grown at a pH of 6**–**8 and 30**–**37 °C and cannot be grown at ≥ 5% NaCl. The optimum pH for growth is 7, and the optimum temperature is 30 °C. Hydrolysis can produce pyruvate, assimilate arabinose, mannitol, and propionate, and use arabinose, glucose, mannose, raffinose, and sucrose to produce acid. The physiological and biochemical details are shown in Table S2.

### Identification of *Microbacterium aurantiacum* 16S rRNA

**Fig. 2:**
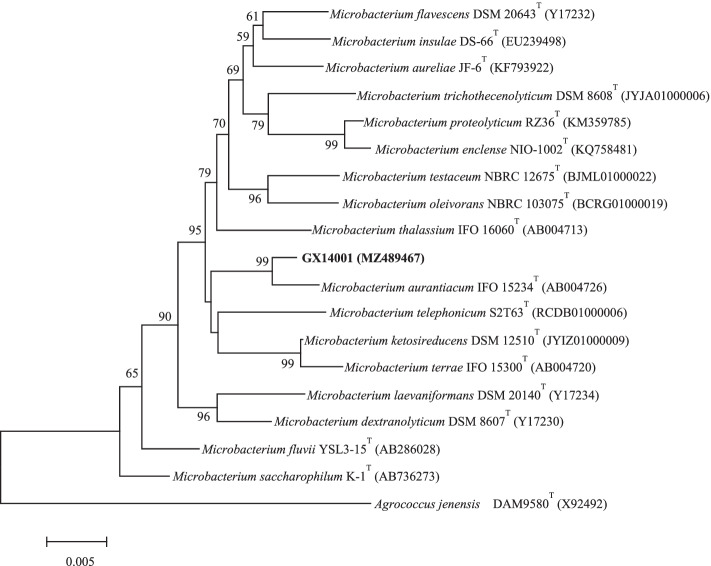
The Neighbor-Joining tree based on the 16S rRNA gene sequences showing the phylogenetic relationship of GX14001 with related taxa. The evolutionary distances were computed using the p-distance method and are in the units of the number of base differences per site. The sequence of *Agrococcus jenensis* DAM9580^T^ (X92492) was used as an outgroup. Bootstrap support values were calculated from 1000 replicates and only values above 50% are shown. Bar, 0.005 substitutions per nucleotide position. Evolutionary analyses were conducted in MEGA6 [23]

The 16S rRNA gene sequences obtained from the sequencing of GX14001 were compared and analyzed on the Ezbiocloud website, and a phylogenetic tree was constructed using MEGA6 [23] (Fig. 2). The outgroup in the phylogenetic tree was selected with reference to the commonly selected outgroup in the *Microbacterium* spp. phylogeny [24]. The results showed that GX14001 had the highest homology and closest genetic relationship with *Microbacterium aurantiacum*; therefore, GX14001 was identified as *Microbacterium aurantiacum*. The sequence of the GX14001 strain was uploaded to NCBI to obtain its GenBank accession number MZ489467.

### Data processing and feasibility analysis

Reads from the down sequencing machine were filtered to obtain high-quality reads. After data filtering, some basic statistics were performed on the data, and the statistics are shown in Table S3. According to Table S3, the Q30s of the downstream data are higher than 93% and the data are available. As shown in Fig. S1, the biological repetition correlation R^2^ values of the control treatment (CK) and GX14001 VOCs treatment (N) were all greater than 0.8, which indicates a high similarity of expression patterns between samples among treatments and good replication of samples.

### Identification of DEGs and functional annotation for differential expression gene analysis of transcriptome samples

**Fig. 3:**
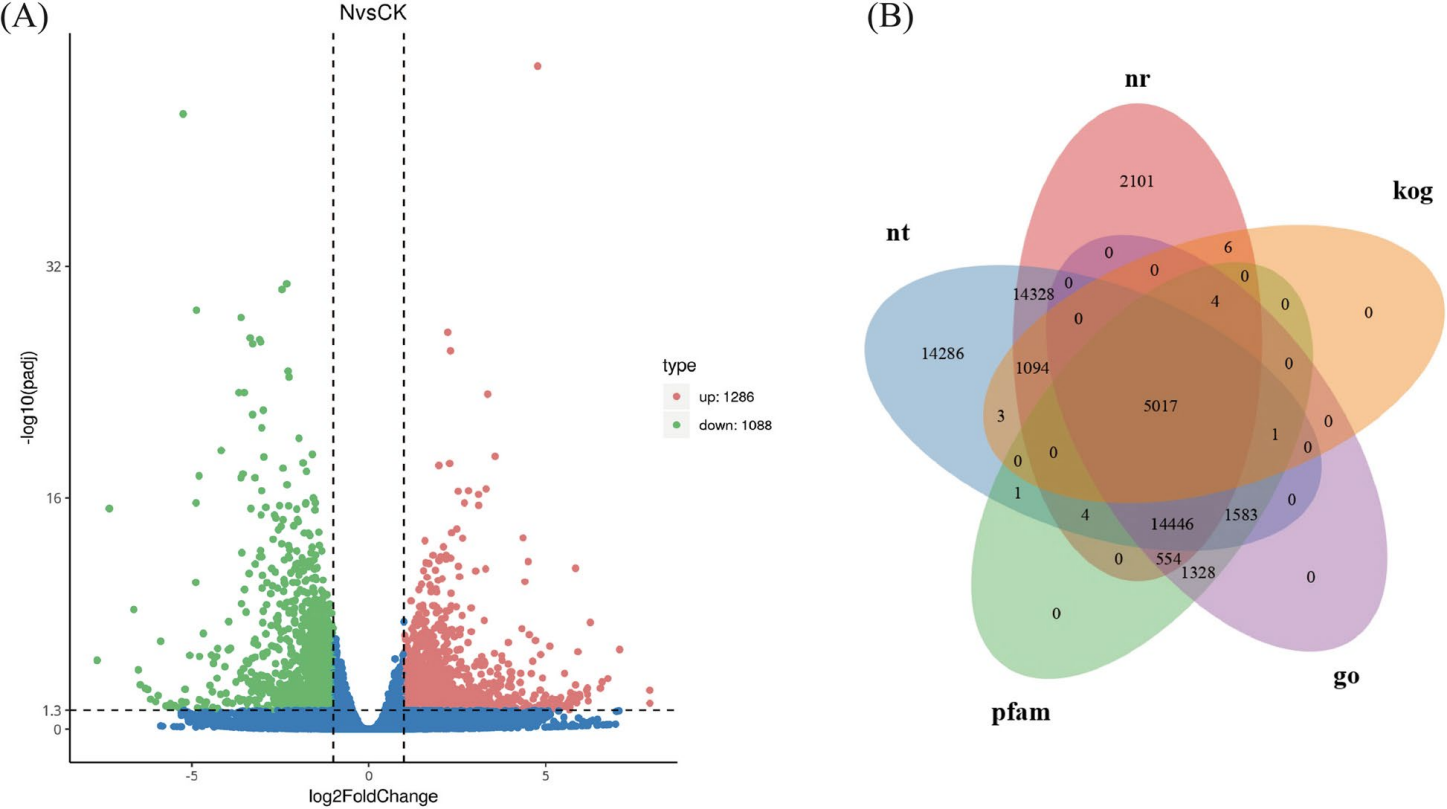
**A** Differential gene expression profile; “N” was treatment group, “CK” was control group. **B** Results of gene function annotation

GOseq and KOBAS software were used for GO functional enrichment analysis of differential gene sets and KEGG pathway enrichment analysis. Genes with an expression that changed more than twice (*P* ≤ 0.05) between the GX14001 VOCs treatment and the control were considered differentially expressed. In this transcriptome sequencing of tobacco, a total of 71,951 genes were detected. Among them, 2374 differentially significantly expressed genes were screened. Among them, 1286 genes (54.2%) were upregulated and 1088 genes (45.8%) were downregulated (Fig. 3-A). To obtain comprehensive gene function information, gene function annotations of seven databases were performed, namely Nr, Nt, Pfam, KOG/COG, Swiss-prot, KEGG, and GO. We selected the annotation results of five databases for display (Fig. 3-B).

### GO functional enrichment analysis of differential genes

**Fig. 4:**
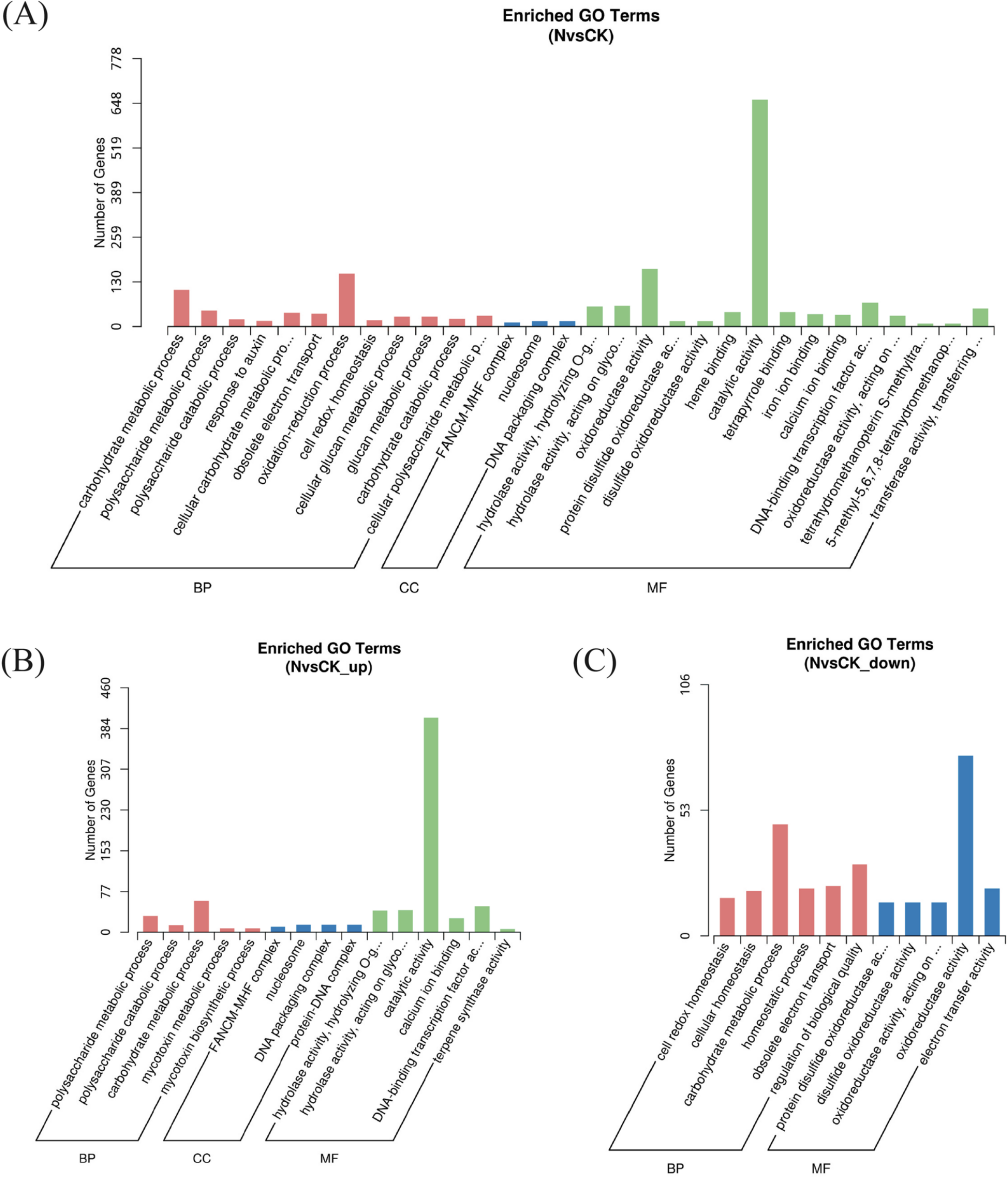
**A** Enrichment Column Diagram of Differential Gene GO; **B** Enrichment Column Diagram of Up-regulated Gene GO; **C** Enrichment Column Diagram of Down-regulated Gene GO. “N” was treatment group, “CK” was control group. BP: biological processes, CC: cellular components, MF: molecular function

The GO enrichment analysis showed that a total of 22,933 genes were enriched, including 1297 differential genes. There were 767 upregulated differential genes and 530 downregulated differential genes in the GO enrichment distribution. The main biological functions performed by the differentially expressed genes from the sequencing results were molecular functions, cellular components and biological processes (Fig. 4).

The sequencing results showed that the upregulated biological processes (BP) were mainly concentrated in polysaccharide metabolism and catabolic processes and carbohydrate metabolism. The upregulated cellular components (CC) were mainly concentrated in the FANCM-MHF complex, nucleosome cellular components, DNA packaging complex and protein. The molecular function (MF) enrichment was focused on hydrolase activity, catalytic activity, calcium ion binding, DNA-binding transcription factor activity, and transferase activity.

The downregulated biological processes were mainly concentrated in cellular homeostasis, carbohydrate metabolic, electron transfer and regulation of biological quality processes. The downregulated molecular functions were mainly in oxidoreductase activity and electron transfer activity. There were no differential downregulated genes in cellular components (CC).

### Analysis of differential gene KEGG enrichment

To further understand the influencing factors of tobacco growth promotion under GX14001 VOCs treatment, important biological information, signal transduction pathways and related metabolic pathways were analyzed, and the related genes and transcription factors involved in the metabolic pathways of plant growth were screened.

**Fig. 5:**
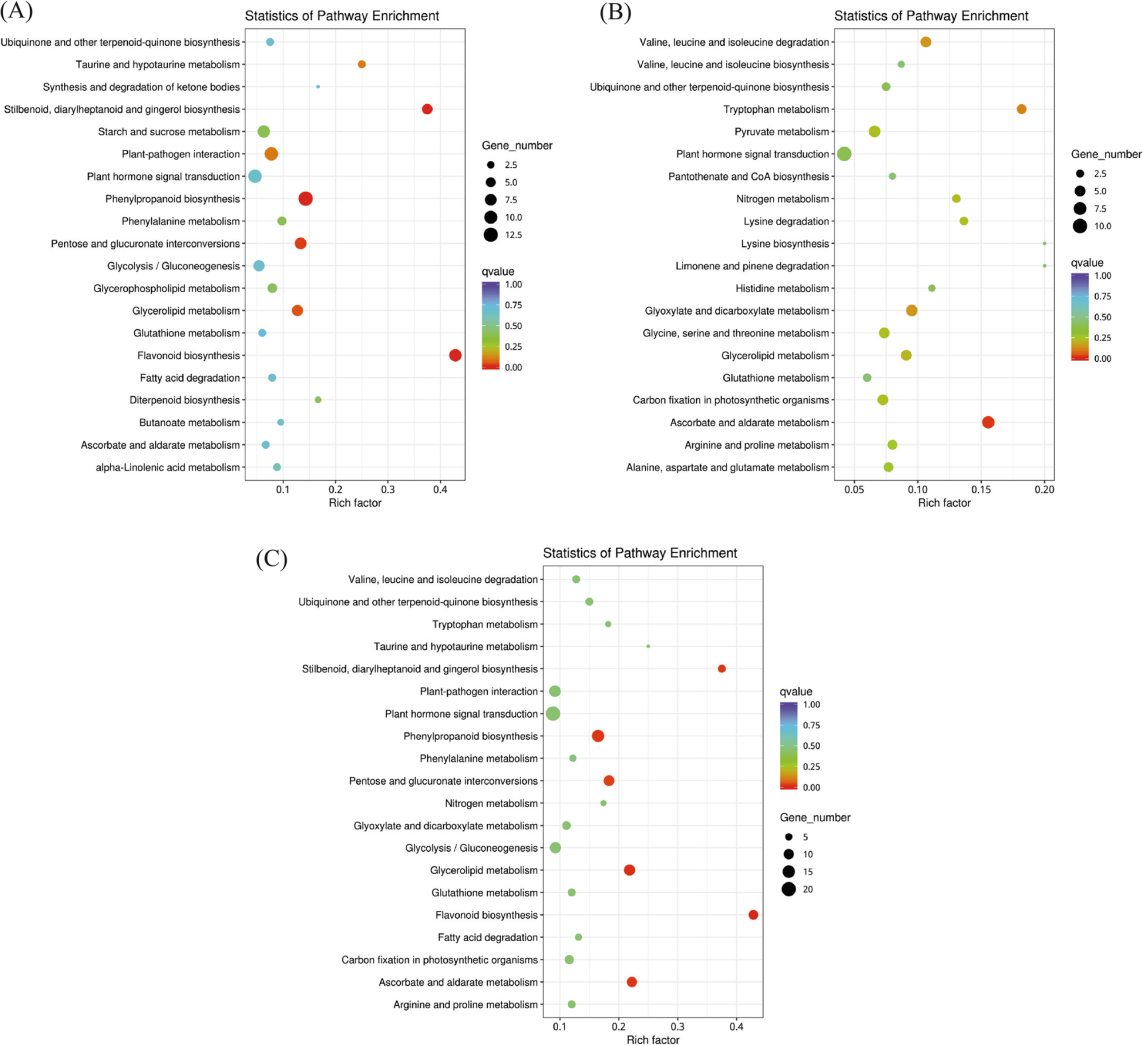
**A** KEGG pathway up-regulated gene top20 enrichment scatter plot; **B** KEGG pathway down-regulated gene top20 enrichment scatter plot; **C** KEGG pathway differential gene enrichment scatter plot

KEGG enrichment analysis was carried out on the data. A total of 63 KEGG upregulated entries and 61 KEGG downregulated entries were enriched. There were 338 differentially expressed genes, 190 of which were upregulated and 148 of which were downregulated (Fig. 5). The enrichment pathways of DEGs were mainly related to “plant hormone signal transduction,” “plant pathogen interaction,” “carbon fixation in photosynthetic organisms,” “stilbene, diphenylheptanone and gingerol biosynthesis,” “phenylpropanoid biosynthesis,” “pentose and glucuronolactone interconversion,” “glyceride metabolism,” “flavonoid biosynthesis” and “ascorbic acid and aldehyde metabolism.”

### qRT-PCR verification of RNA-seq

**Fig. 6:**
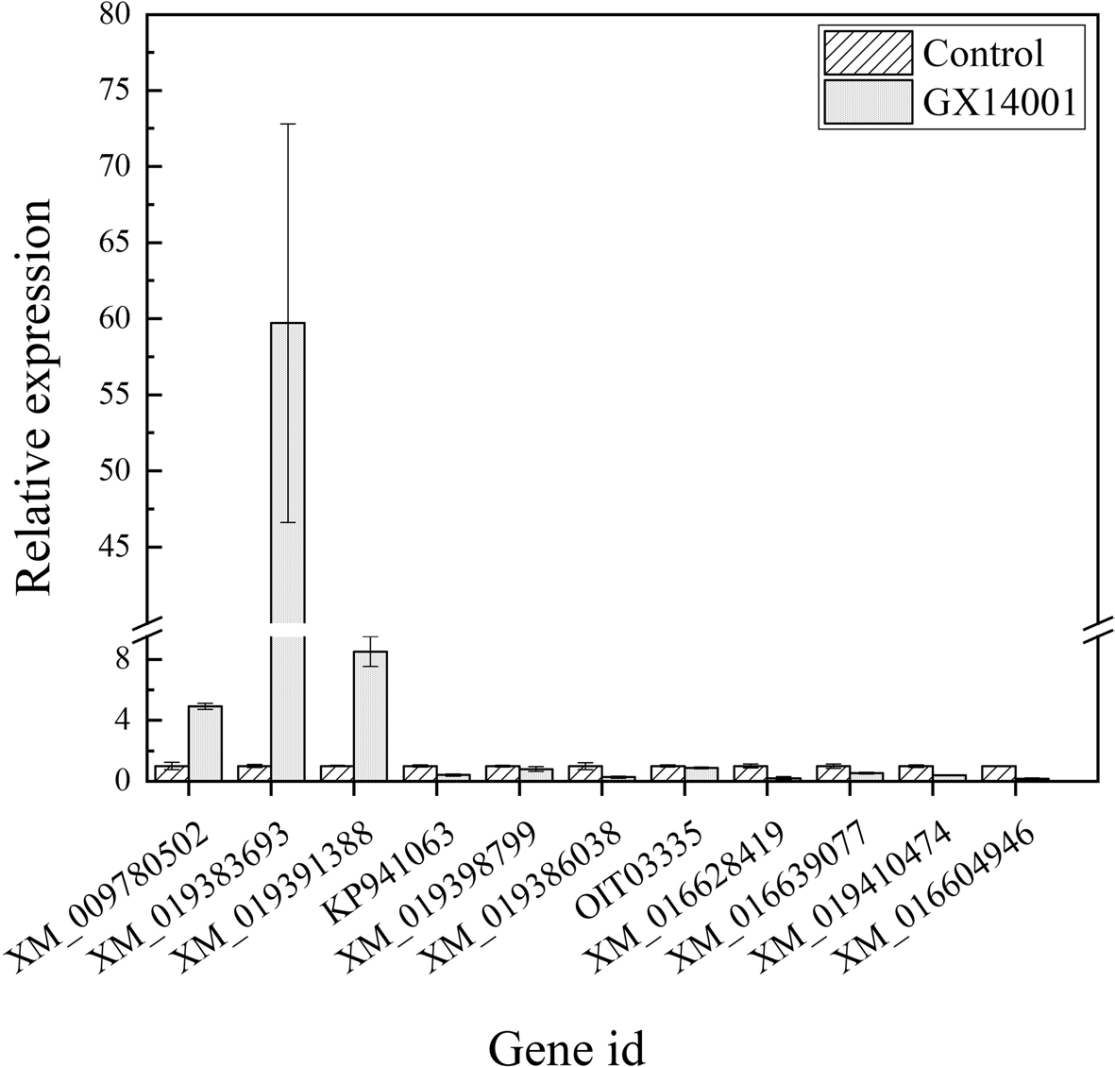
Relative expression levels are shown as induction fold in GX14001-VOCs treatment compared with control. Error bars represent standard deviation from triplicate repeats. XM_009780502, auxin response factor 5, *ARF5*; XM_019383693, ethylene-responsive transcription factor 1B-like, *ERF1*; XM_019391388, *Nicotiana attenuata* protein gene, *TIFY-10A*; KP941063, Auxin responsive gene, *GH3.6*; XM_019398799, *Nicotiana attenuata* regulatory protein gene, *NPR5*; XM_019386038, *Nicotiana attenuata* auxin-responsive protein gene, *SAUR50*; OIT03335, auxin-responsive protein gene, *SAUR32*; XM_016628419, *Nicotiana tabacum* histidine protein kinase gene, *HPK3*; XM_016639077, XM_019410474, XM_016604946, *Nicotiana tabacum* auxin-induced protein gene, *AIP5*.

According to GO functional annotation and KEGG metabolic pathway analysis, 11 genes in the plant hormone signaling pathway (XM_009780502, auxin response factor 5, ARF5; XM_019383693, ethylene-responsive transcription factor 1B-like, ERF1; XM_019391388, Nicotiana attenuata protein gene, TIFY-10A; KP941063, auxin responsive gene, GH3.6; XM_019398799, Nicotiana attenuata regulatory protein gene, NPR5; XM_019386038, Nicotiana attenuata auxin-responsive protein gene, SAUR50; OIT03335, auxin-responsive protein gene, SAUR32; XM_016628419, Nicotiana tabacum histidine protein kinase gene, HPK3; XM_016639077, XM_019410474, XM_016604946, Nicotiana tabacum auxin-induced protein gene, AIP5) were screened and verified by qRT-PCR. The results showed that the genes of XM_009780502, XM_019383693 and XM_019391388 were upregulated, and the genes of KP941063, XM_019398799, XM_019386038, OIT03335, XM_016628419, XM_016639077, XM_019410474 and XM_016604946 were downregulated. The expression trend obtained in the qRT-PCR analysis was consistent with that in the transcriptome (Fig. 6).

### Study of plant hormone signaling pathways

**Fig. 7:**
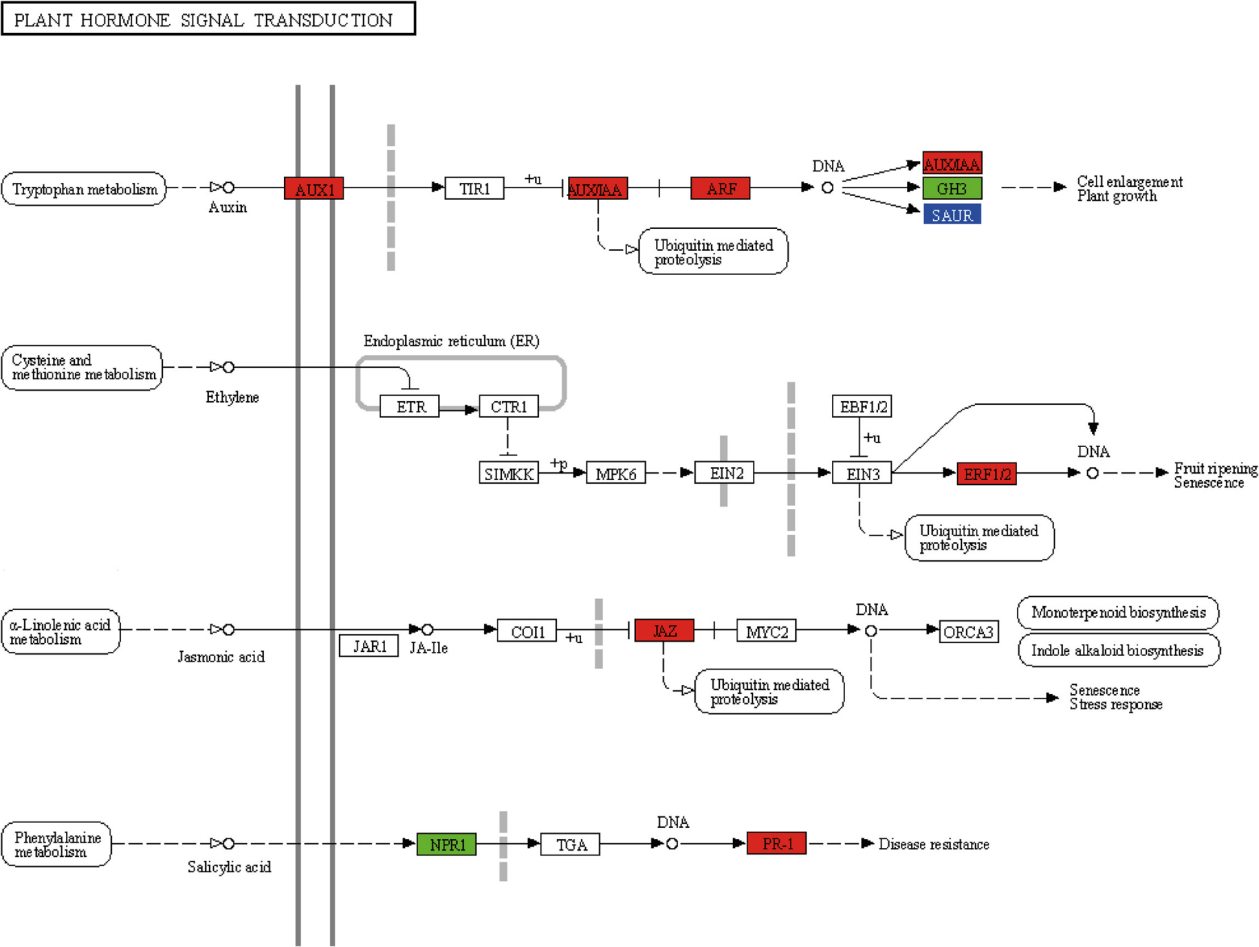
Plant hormone signal transduction pathways. The colors in the figure represent differentially significant genes. Red shows genes that are significantly upregulated. Green shows genes that are significantly downregulated. Blue indicates that there are both significantly up-regulated and down-regulated genes

The transcriptome sequencing results of tobacco co-cultured with GX14001 VOCs for two weeks showed that the differentially expressed genes were mainly in the plant hormone signal transduction pathway (Fig. 7, which obtained by KEGG [25]). These hormone pathways included auxin, cytokinin, gibberellin, ethylene, jasmonic acid, and salicylic acid. These hormones are not only involved in the regulation of plant growth and development but can also be used as key factors mediating the plant stress response.

The results of I-plate experiments showed that treatment with GX14001 VOCs significantly promoted the growth of tobacco, with significant increases in leaf expansion, fresh weight, root length, and lateral root number (*P* < 0.01). Transcriptome data showed that in the auxin signaling pathway, the expression of auxin transporter gene *AUX1*, auxin positive regulatory response factor *IAA27*, auxin response factor *ARF5*, and the genes encoding tobacco auxin-induced protein X15 in the SAUR family were all upregulated by roughly 2.02, 2.04, 2.28, and 4.02 times, respectively. The downregulated expression of *SAUR32* and *SAUR50* may induce plant growth by regulating cell wall acidification, and the expression of *SAUR32* and *SAUR50* was downregulated by 2.5 and 4.5 times, respectively; *GH3*.*6* may be downregulated in response to the regulation of other plant hormone signaling pathways, with downregulation by 3.7 times. In gene expression of the ethylene signal transduction pathway, the *ERF1* transcription factor regulates the ethylene response gene, and expression was upregulated by 14.16 times. Differentially expressed genes in the jasmonic acid signaling pathway, annotated to the *TIFY* family XM_009794995 and XM_019391388, are key genes in jasmonic acid signal transduction, which can enhance plant stress resistance and upregulate expression, and the different multiples are 3.02 and 6.04. In the salicylic acid signaling pathway, the expression levels of XM_019368642 and X52555 genes in the disease-related *PR1* gene family were upregulated, and the differences were 10.52 and 12.36, respectively, indicating that tobacco treated with GX14001 VOCs have improved disease resistance.

### Effects of GX14001 VOCs on the growth of *Arabidopsis mutants*

**Fig. 8:**
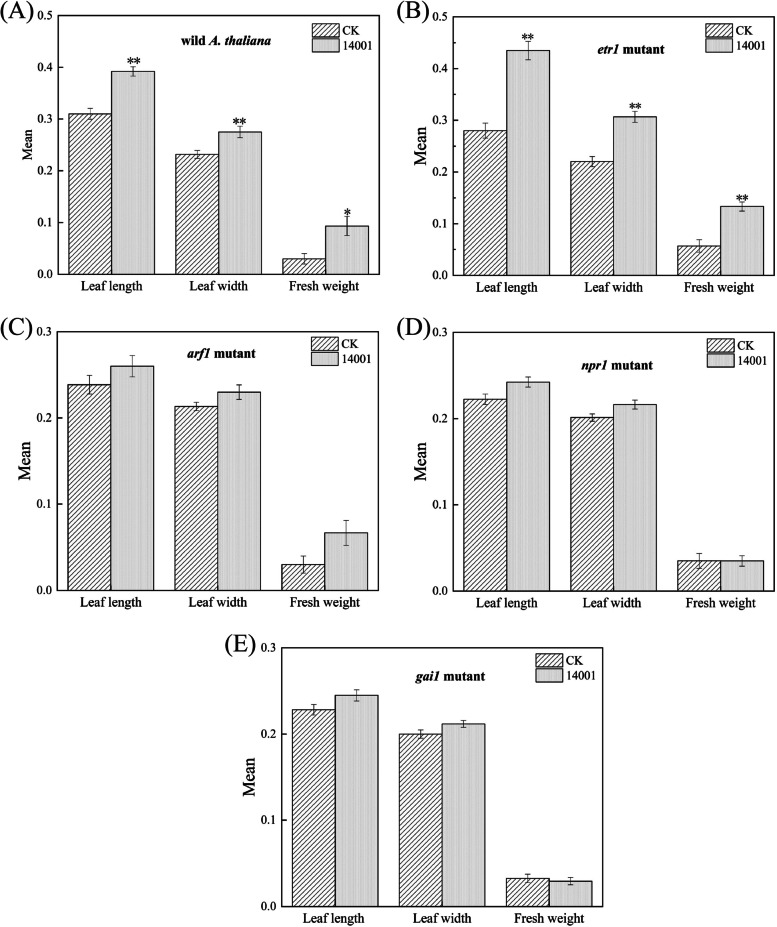
**A** Growth-promoting effect of strain GX14001 on wild *A. thaliana*; **B** Growth-promoting effect of strain GX14001 on *etr1* mutant; **C** Growth-promoting effect of strain GX14001 on *arf1* mutant; **D** Growth-promoting effect of strain GX14001 on *npr1* mutant; **E** Growth-promoting effect of strain GX14001 on *gai1* mutant. "*" refers to significant difference between treatments,* P* < 0.05, "**" refers to *P* < 0.01. The unit of leaf length and width and root length is cm; the unit of fresh weight is g

To further investigate the mechanism of VOCs produced by strain GX14001 promoting plant growth, we used *Arabidopsis* mutants to explore the role of plant hormone signaling in GX14001 VOCs promoting *Arabidopsis* growth. The results showed that GX14001 VOCs had significant growth-promoting effects on the leaves and fresh weight of the *Arabidopsis* wild type and ethylene (*etr1*) *Arabidopsis* mutant (Fig. 8 A, B), while there were no significant growth-promoting effects on auxin (*arf1*), salicylic acid (*npr1*), and gibberellin (*gai1*) (Fig. 8 C, D, E). This indicated that the growth-promoting effect of strain GX14001 VOCs on plants mainly depended on auxin, salicylic acid, and gibberellin pathways, rather than the ethylene pathway (Table 1).

**Table 1:** Growth promotion response of *A. thaliana mutants* to VOCs

*A.thaliana mutants*	Description	Growth promoting effect of GX14001-VOCs
*etr1*	Ethylene insensitivity	+
*arf1*	Auxin receptor deficiency	-
*npr1*	Salicylic acid receptor deficiency	-
*gai1*	Gibberellin insensitive	-

“ + ”means that the strain has plant growth promotion,“-”means that the strain has no plant growth promotion

## Discussion

The *Microbacterium* sp. has been identified in previous studies as an important class of phospholysis bacteria [26–28]. The application of *Microbacterium* sp. in agriculture can also be traced back to 2007 when Karlidag et al. (2007) found that the combination of *Microbacterium* FS01 and *Bacillus* M3 or OSU-142 had the potential to improve yield, growth, and nutrition in apple trees [29]. Sheng et al. (2008) isolated *Microbacterium* G16 from rape roots in heavy metal contaminated soil and found that inoculation with G16 increased rape biomass and total lead uptake, while G16 exhibited different multiple heavy metal and antibiotic resistance characteristics and increased water-soluble lead in solution and soil [30]. Cordovez et al. (2018) reported that the volatiles of *Microbacterium* strain EC8 promote plant growth by regulating sulfur and nitrogen metabolism through roots and found that its induction of plant growth promotion was tissue specific [31]. Therefore, *Microbacterium* sp. has certain prospective applications in agriculture, but no previous studies have been conducted on *Microbacterium aurantiacum* in this regard.

In this study, we found for the first time that VOCs produced by *Microbacterium aurantiacum* can significantly promote the growth of tobacco. We analyzed the transcriptome of differentially expressed genes between the treated group of *Nicotiana benthamiana* that had been inoculated with GX14001 VOCs and the non-inoculated group as a control contrast, and we found that its VOCs may exert their pro-growth effects mainly through the plant hormone signaling pathway. Numerous studies have shown that most plant hormones play a complex role in regulating the growth and development of plants. The same hormone can regulate multiple development processes, and the same given development process requires the synergistic action of many different hormones. As found in this study, downregulated expression of *ARR-17*, a two-component response regulator gene in the cytokinin pathway, promoted cell division and bud germination; and key genes in the auxin, ethylene, jasmonic acid, and salicylic acid pathways had significantly upregulated expression, promoting all aspects of tobacco growth. In addition, due to the mutual crosstalk between plant hormone signals, different hormone signals act differently on each other and thus have different effects on plant growth and development. For example, Traw et al. (2003) found that jasmonic acid, salicylic acid, and gibberellin interacted in the induction of trichomes in *Arabidopsis*, with jasmonate and salicylate-dependent exhibited antagonistic effects, while jasmonic acid and gibberellin interacted synergistically [32]. Kong et al. (2020) found that auxin and salicylic acid signals could antagonize each other in the lateral root development of *Arabidopsis* [33]. Ghorbel et al. (2021) proposed the idea that the existence of mutual antagonism between cytokinin and jasmonic acid during xylem growth and development of *Arabidopsis* roots [34]. In this study, the expression of *AHK2_3_4*, a cytokinin receptor gene encoding a regulatory histidine kinase in the cytokinin pathway, an *ERF5* transcription factor in the gibberellin pathway, and *NPR5-like* gene encoding a regulatory protein in the salicylate pathway were all downregulated; expression of the *DELLA-GAI* protein gene, which negatively regulates the gibberellin synthesis pathway, was upregulated, and it is speculated that the expression of these genes may respond to multiple signaling pathways, but we are not certain of its specific mechanism at present. Subsequent experimental validation by *Arabidopsis* mutants revealed that the growth-promoting effect may depend mainly on three pathways, auxin, gibberellin, and salicylic acid pathways, but not on ethylene or other pathways. Finally, we found that key genes in phenylpropanoid biosynthesis, flavonoid biosynthesis, stilbenoid, diarylheptanoid, and gingerol biosynthesis pathways were differentially expressed and significantly upregulated, and it was hypothesized that the upregulated expression of these genes increased the accumulation of flavonoids, astragalus compounds, and lignin, thus promoting the growth and development of tobacco.

Our studies only expound on the growth-promoting effect and mechanism of VOCs produced by *Microbacterium aurantiacum* on tobacco; the VOCs produced by this strain were not explored, which will be further investigated subsequently. In addition, up to now, some volatile organic compounds are being studied or used in agriculture, for example, studies have proved that 2-nonanone, 2-undecanone, and 2-tridecanone released by solid lipid nanoparticles and nanostructured lipid carriers can stimulate the growth induction of L. *sativa* and *S. lycopersicum* by improving the development of shoot and root; Novellus (based on terpene compounds), a biological bactericide produced by Eden Research, UK, can effectively control grey mold (*Botrytis cinerea*) on wine grapes and fresh grapes. But on the whole, there are some difficulties in the application of volatile organic compounds in agriculture, which may be due to the following reasons. First, most of the studies on the promotion of plant growth by VOCs produced by strains have been tested in laboratory culture dishes, but have not been applied in the field. Second, in the practical application of agriculture, there are also questions as to whether the strain can colonize the soil and the stability of the VOCs produced by it to promote plant growth. Finally, the application of VOCs must consider the complexity of actual agricultural environmental conditions. Studies have shown that VOCs are extremely unstable in polluted environments [35]. Technical limitations and defects are the key problems we must solve in the future.

## Materials and methods

### Pretreatment of tobacco and *Arabidopsis* mutants

Tobacco and *A*. *thaliana* (ecotype Col-0) were studied, which were obtained from our lab (Guangxi Key Laboratory for Polysaccharide Materials and Modifications, School of Marine Sciences and Biotechnology, Guangxi Minzu University). The phytohormone biosynthesis and metabolism-related gene mutants (*etr1*, *arf1*, *npr1* and *gai1*) were obtained from the Arabidopsis Biological Resource Center (http://www.arabidopsis.org) or the European Arabidopsis Stock Centre (http://arabidopsis.info). The required tobacco / *Arabidopsis* mutant seeds were placed in a 1.5 mL Eppendorf (EP) tube. The test plant seeds were surface sterilized by soaking in 75% (V/V) ethanol for 2–3 min, followed by soaking in 1% sodium hypochlorite solution for 2–3 min, and then soaking in sterilized water 4–5 times until the disinfectants were completely removed to avoid its influence on seed development. Seeds in EP tubes were placed on sterilized filter paper and dried in an ultra-clean worktable. Seeds were plated on Murashige and Skoog Basal Medium (MS medium) [36] with sterilized bamboo sticks. After completion, the plate was sealed with sealing film and cultivated in a 25 °C intelligent artificial climate box until germination. The conditions of the intelligent artificial climate box were light 16 h (140 µmol photons m^−2^ /s), dark 8 h, and temperature 25 °C.

### Isolation and identification of strains and fermentation broth

Strain GX14001 was isolated from the intestinal tract of fish (not live fish) by our lab and collected from Beihai, Guangxi, China. The strain GX14001 has been preserved in China General Microbiological Culture Collection Center with the preservation number of CGMCC 1.19276. In this study, the taxonomic status of isolate GX14001 was determined by phylogenetic analysis and physiological characteristics. The physiological characteristics refer to the method reported by Niste et al. [37]. The tested bacteria were used to inoculate Trypticase Soy Broth (TSB) liquid medium and cultured at 30 °C and 200 r/min for 24 h. Subsequently, bacteria were diluted with sterile water to about 1 × 10^8^ CFU/mL.

Special statement: The samples used in the experiment were not taken from live fish.

### Co-culture experiment

The experiment was carried out on a 9 cm plate divided into two sections (I-plate). One side was used to culture tobacco, and the other side was used to culture bacteria. Due to the limitations of the I-plate, bacteria cannot contact and communicate directly with tobacco, while the VOCs produced by bacteria can communicate and interact with tobacco through the upper part of the culture plate (refer to the method reported by Zhang et al. 2007) [3]. The MS medium was used on the plant side, and Tryptose Soya Agar Medium (TSA medium) was used for the bacteria. After 5 d of tobacco culture, the tobacco seedlings with the same growth conditions were transplanted to the side of the two-cell culture plate MS, and 5 seedlings were transplanted in each culture plate to maintain the same spacing of tobacco plants. A 10 μL bacterial suspension was used to inoculate the other side of the TSA medium, and the bacterial content was about 1 × 10^8^ CFU/mL. The control experiment was inoculated on TSA medium with 10 μL sterile water, and the treatment group and the control group did three replicates respectively. After that, the culture dishes were sealed with sealing parafilm and cultured in an intelligent artificial climate chamber. The culture conditions were consistent with those used previously. After two weeks of culture, the biomass of tobacco, namely leaf length and width, root length, lateral root number and fresh weight was measured or counted.

### Plant transcriptome analysis

Three biological replicates (plates) of each group of control and treatment group were sent to Wekemo Tech Co., Ltd. Shenzhen China for transcriptome sequencing analysis. The treatment group was tobacco plants exposed to volatile substances of strain GX14001 for two weeks.

### RNA extraction, library construction and RNA-Seq

Total RNA was extracted from the control groups CK_1, CK_2, and CK_3 and the treatment groups N_1, N_2, and N_3. The experiment was divided into two experimental groups, each with three replicates (non-treated with bacteria (control group: CK_1, CK_2, CK_3) and bacterial-treated with GX14001 VOCs (treatment group: N_1, N_2, N_3). The quality and quantity of total RNA were accurately detected by an Agilent 2100 bioanalyzer. Briefly, mRNA was purified from total RNA using poly-T oligo-attached magnetic beads. Fragmentation was carried out using divalent cations under an elevated temperature in the first strand synthesis reaction buffer (5X). First strand cDNA was synthesized using a random hexamer primer and M-MuLV reverse transcriptase (RNase H-). Second strand cDNA synthesis was subsequently performed using DNA polymerase I and RNase H. The remaining overhangs were converted into blunt ends via exonuclease/polymerase activities. After adenylation of the 3’ ends of DNA fragments, adaptors with hairpin loop structures were ligated to prepare for hybridization. To select cDNA fragments of preferentially 370**–**420 bp in length, the library fragments were purified with the AMPure XP system (Beckman Coulter, Beverly, USA). Then, PCR was performed with Phusion High-Fidelity DNA polymerase, Universal PCR primers, and the Index (X) Primer. Finally, PCR products were purified (AMPure XP system), and library quality was assessed on the Qubit2.0 Fluorometer, Agilent Bioanalyzer 2100 systems and qRT-PCR. After the library was qualified, the cDNA library was sequenced on the Illumina Novaseq 6000 platform. Three biological replications were used for RNA-seq experiments.

### Differential expression analysis

The clean data (clean reads) were obtained by removing reads containing an adapter, reads containing ploy-N, and low**-**quality reads from raw data. At the same time, the Q20, Q30 and GC content of the clean data were calculated. All downstream analyses were based on high-quality clean data. After getting clean reads, Trinity was used to splice clean reads [38]. The transcript sequence obtained by Trinity splicing was used as a reference sequence for subsequent analysis. The longest cluster sequence was obtained after hierarchical clustering by Corset for subsequent analysis [39]. Differential expression analysis for both groups was performed using the DESeq2 R package (1.20.0). DESeq2 provides statistical procedures for determining differential expression in numerical gene expression data using a model based on a negative binomial distribution. The obtained p-values were adjusted to the control to determine the false discovery rate using the method of Benjamini and Hochberg [40]. The genes with adjusted *P* value < 0.05 found by DESeq2 were designated as differentially expressed genes. The GO function enrichment analysis and KEGG pathway enrichment analysis were performed by GOseq and KOBAS software.

### qRT-PCR validation

To validate the RNA-seq data, expression levels of 11 DEGs selected randomly were screened by qRT-PCR, and actin and glyceraldehyde-3-phosphate dehydrogenase (GAPDH) were selected as internal control genes. As mentioned above, RNA was isolated from the control group CK_1, CK_2, and CK_3, and the treatment group, N_1, N_2, and N_3. Reverse transcription was conducted using the StarScript II First-strand cDNA Synthesis Kit (GenStar, Beijing, China). The gene primers and the internal control for qRT-PCR are listed in Table S1. All primers were synthesized by AuGCT DNA-SYN Biotechnology Company (Wuhan, China) and tested by regular PCR. The primer concentration was 10 μM, and the DNA quantity was 200 ng/μL. The specificity of the product was checked by 2% agarose gel electrophoresis prior to the melt curve. The qRT-PCR system comprised 5 μL EvaGreen 2 × qPCR MAsterMix, 0.3 µL of each primer, 1 µL diluted cDNA, and 3.4 µL DNase/RNase-free ddH_2_O. PCR amplification was conducted with the following conditions: 1 cycle of 95℃ for 10 min, 40 cycles of 95℃ for 15 s, and 60℃ for 60 s.

### Data Processing

Microsoft Excel 2016 and IBM SPSS Statistics 21 software were used for statistical analysis of the data. One-way analysis of variance and LSD were used for the significant difference test. OriginPro 2018 was used for histogram plotting, and the relative expression levels of the DEGs were calculated using the 2^−ΔΔ Ct^ method [41].

## Supplementary Information

**Additional file 1:** **Table S1. **Primers used for Real-Time PCR. **Table S2. **Physiologicaland biochemical characteristics of GX14001. **Table S3.** QC Reads quality results. **Fig S1.** Correlation Heat Map of tobacco TranscriptomeSamples.

## Data Availability

Raw reads have been deposited in the National Center for Biotechnology Information (NCBI) and received BioProject ID PRJNA761523. The data will be accessible with the following link: “ https://www.ncbi.nlm.nih.gov/bioproject/PRJNA761523”. We have obtained permission to collect *Arabidopsis thaliana.*

## Abbreviations

PGPR: Plant Growth Promoting Rhizobacteria
VOCs: Volatile Organic Compounds
GO: Gene Ontology
16S rRNA: 16S ribosomal RNA
ABA: Abscisic Acid
IAA: 3-Indoleacetic Acid
SAR: Systemic Acquired Resistance
ISR: Induced Systemic Resistance
JA: Jasmonic Acid
ET: Ethylene
IST: Induced Systemic Tolerance
BP: Biological Processes
CC: Cellular Components
MF: Molecular Function
DEG: Differentially Expressed Gene
KEGG: Kyoto Encyclopedia of Eenes and Eenomes
EP tube: Eppendorf tube
Tobacco: *Nicotiana benthamiana*
