# Supplementary material for: Transcriptome analysis of the growth-promoting effect of volatile organic compounds produced by Microbacterium aurantiacum GX14001 on tobacco (Nicotiana benthamiana)
Source: BMC Plant Biol. 2022 Apr 22;22:208. doi: 10.1186/s12870-022-03591-z (PMC9028074; doi:10.1186/s12870-022-03591-z)
Supplement: Supplementary file 1 — Additional file 1: Table S1. Primers used for Real-Time PCR. Table S2. Physiologicaland biochemical characteristics of GX14001. Table S3. QC Reads quality results. Fig S1. Correlation Heat Map of tobacco TranscriptomeSamples. [file 12870_2022_3591_MOESM1_ESM.docx]

**Journal name：**BMC Plant Biology

**Manuscript Title：**Transcriptome analysis of the growth-promoting effect of volatile organic compounds produced by *Microbacterium aurantiacum* GX14001 on tobacco (*Nicotiana benthamiana*)

**-The name(s) of the author(s)**

**-The affiliation(s) and address(es) of the author(s)**

**-The e-mail address, telephone and fax numbers of the corresponding author**

Yahui Gao^1^†, Jing Feng^1^†, Jiafa Wu^1^, Kun Wang^1^, Shuang Wu^1^, Hongcun Liu^1^, Mingguo Jiang^1*^

^1^ *Guangxi Key Laboratory for Polysaccharide Materials and Modifications, School of Marine Sciences and Biotechnology, Guangxi Minzu University, Nanning 530008, China*

† *These authors contributed equally to this work*

^*^Corresponding author: Mingguo Jiang; E-mail: mzxyjiang@163.com.

**Supplementary data:**

1. Primers used for Real-Time PCR

| **Table S1 Primers used for Real-Time PCR** | |
| --- | --- |
| Gene ID/name | Primer sequence（5’-3’） |
| Actin | CTACTTACTGAAGCACCCTTGAATCC |
|  | CCTGCCCATCTGGTAACTCATAGC |
| GAPDH | CTGCTCACTTGAAGGGTGGT |
|  | GGGAGCAAGGCAATTTGTGG |
| XM_009780502 | GAAACGGAATTATCAATCACC |
|  | CTTTTCCCAAACAAACCTTC |
| XM_019383693 | CATGTCACTGCATTACAAGA |
|  | GATTTTCCTAAAGTTGCACCA |
| XM_019391388 | GATATTGGCACGTGAGTTCGC |
|  | TCTGAACCAGAAAAGGCACA |
| KP941063 | CCTGATTTACACAAAGGCAA |
|  | AGAATTGTTTCATTTGGGCTA |
| XM_019398799 | TCTTGATGAATCGCTCGCATT |
|  | GATCCAATAGAACCGCCACC |
| XM_019386038 | CTGCAGCTTGTGACAACTTGT |
|  | CCTCCTTGCCCCTTCACTTT |
| OIT03335 | TTAAAGCTTCCGTGCTCCGT |
|  | AAGAGCGGAAGGACAAAGCA |
| XM_016628419 | ATTTACCAAAGTTGCGCCCG |
|  | TGGTCCCAAGCAAGTTGTGA |
| XM_016639077 | AAGAATTTGGCTTCTATCATCC |
|  | TCTCCTGCTTTGCTATACGAA |
| XM_019410474 | AGCATATGGAATCTTACCGAG |
|  | GAACTTAACTCCACAAAGCCT |
| XM_016604946 | TAGTTTCACTTCACATCCCT |
|  | CTTGTTTGATTTTCTTATGGC |

2. Physiological-biochemical identification of *Microbacterium aurantiacum*.(Table S2)

| **Table S2 Physiological and biochemical characteristics of GX14001** | |
| --- | --- |
| Characteristic | GX14001 |
| Colony colour | Orange |
| Motility | - |
| Growth at 37℃ | + |
| Growth in 2% NaCl | + |
| Growth in 5% NaCl | - |
| Growth in 6% NaCl | - |
| Hydrolysis (API 20E) |  |
| Gelatin | - |
| Starch | - |
| H_2_S formation | - |
| VP test | + |
| Assimilation of (API 20NE) |  |
| Arabinose | + |
| Maltose | - |
| Mannose | - |
| Mannitol | + |
| N-Acetylglucosamine | - |
| Acetate | - |
| Adipate | - |
| Caprate | - |
| Citrate | - |
| Fumarate | - |
| Gluconate | - |
| Lactate | - |
| Malate | - |
| Phenyl acetate | - |
| Propionate | + |
| Acid produced from (API 50CH) |  |
| L-Arabinose | + |
| Galactose | - |
| Glucose | + |
| Inulin | - |
| Mannose | + |
| Melezitose | - |
| Raffinose | + |
| Rhamnose | - |
| Ribose | - |
| Sucrose | + |
| Trehalose | - |
| Xylose | - |

3. Transcriptome data quality control

The reads from the sequencing down machine are filtered to get high quality reads. after data filtering, we do some basic statistics on the data, and the statistics are shown in Table S3. Each base measured will give a corresponding quality value, and this quality value is a measure of sequencing accuracy. Q20 and Q30 indicate the percentage of bases with quality values greater than or equal to 20 or 30. Generally, the minimum Q30>85% is required to measure the quality control data of the downstream data. According to Table S3, the Q30 of the downstream data are higher than 93% and the data are available.

| **Table S3 QC Reads quality results** | | | | | | | |
| --- | --- | --- | --- | --- | --- | --- | --- |
| Sample | Raw Reads | Clean Reads | Clean Bases | Error(%) | Q20(%) | Q30(%) | GC Content(%) |
| N_1 | 22862523 | 22067766 | 6.62G | 0.03 | 97.7 | 93.22 | 42.47 |
| N_2 | 24282457 | 23006161 | 6.90G | 0.03 | 97.84 | 93.59 | 42.76 |
| N_3 | 22348662 | 21543593 | 6.46G | 0.03 | 97.69 | 93.18 | 42.57 |
| CK_1 | 23094895 | 22079037 | 6.62G | 0.03 | 97.93 | 93.75 | 42.55 |
| CK_2 | 23198799 | 22320833 | 6.70G | 0.02 | 98.39 | 94.72 | 42.76 |
| CK_3 | 20928271 | 20008089 | 6.00G | 0.02 | 98.36 | 94.65 | 42.72 |
| Raw Reads: the data volume of the original sequence data; Clean Reads: the amount of filtered sequencing data; Clean Bases: the number of sequencing sequences multiplied by the length of sequencing sequences, and converted to g as the unit; Error(%): sequencing base error rate; Q20 (%): The percentage of bases with pH red value greater than 20 in the total base. Q30 (%): the percentage of bases with pH red value greater than 30 in the total base; GC Content(%): the percentage of the total number of bases G and C in the total number of bases. | | | | | | | |

4. Correlation analysis of transcriptome samples

| 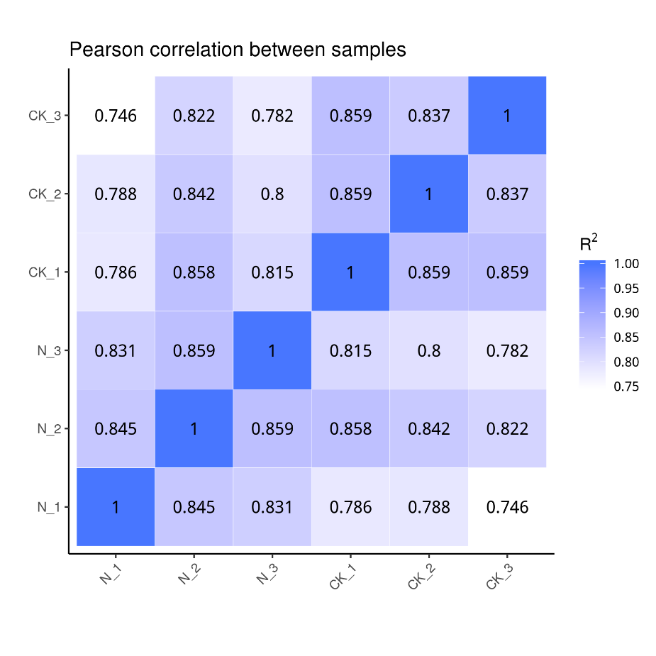 |
| --- |
| Fig.S1 Correlation Heat Map of tobacco Transcriptome Samples |
